# Supplementary material for: Meta-analysis of public raw sequence data unveils the distribution and dynamics of emerging aquatic pathogens: using Macrobrachium rosenbergii golda virus as a case study
Source: Microbiol Spectr. 2026 Feb 9;14(3):e02869-25. doi: 10.1128/spectrum.02869-25 (PMC12955490; doi:10.1128/spectrum.02869-25)
Supplement: Supplemental figures — Fig. S1 and S2. [file spectrum.02869-25-s0001.docx]

**
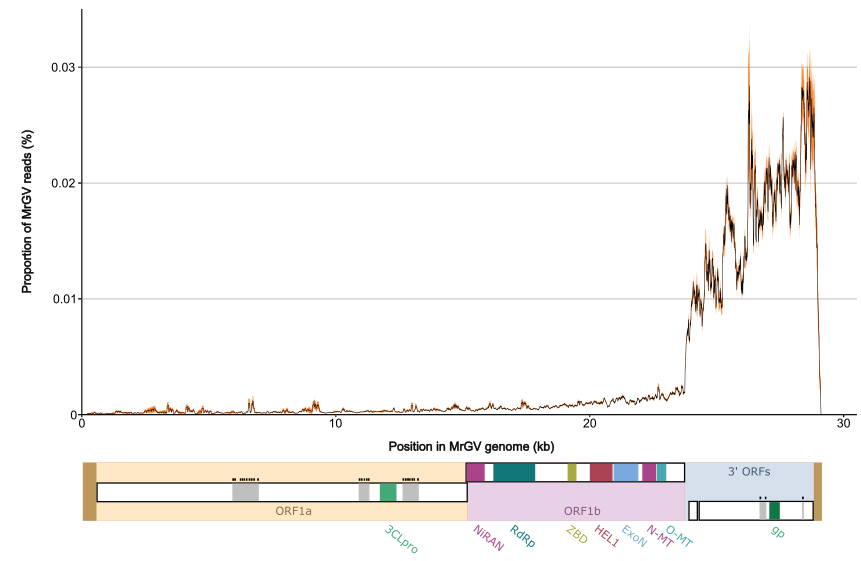
Supplementary figure 1:** Graph showing the location of mapped reads across the MrGV genome from all SRAs (*n* = 145). Orange ribbon represents the 90% confidence interval.


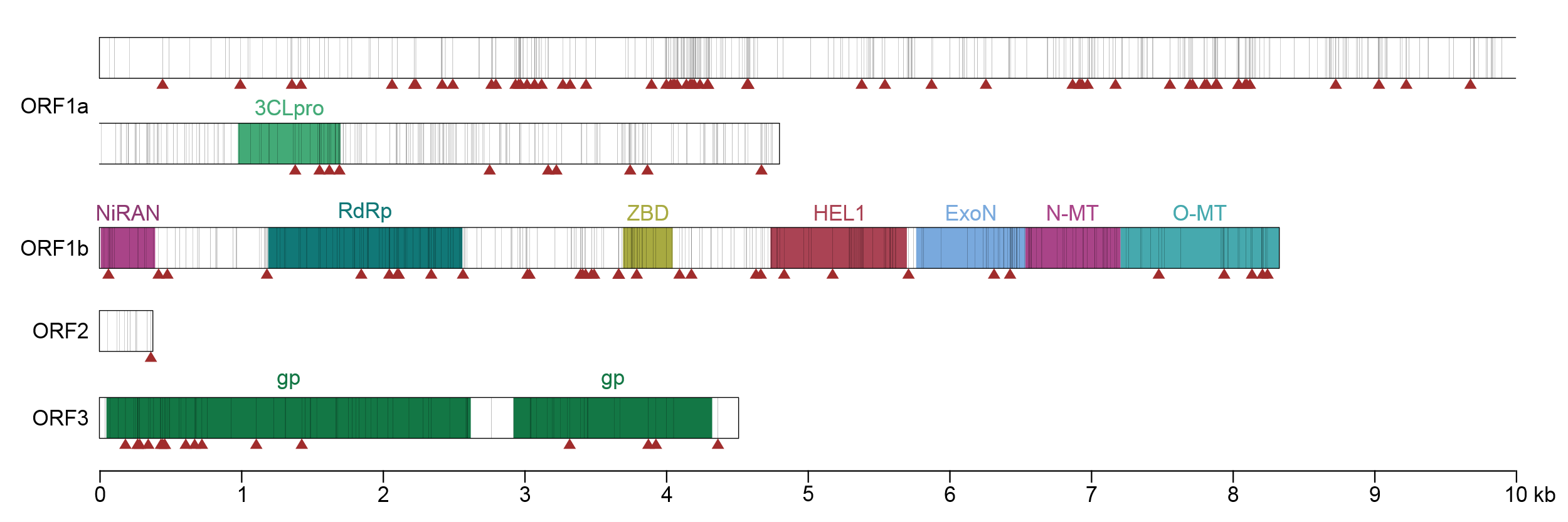


**Supplementary figure 2:** Location of sites within the coding regions of the MrGV genome that had a nucleotide change in at least one full MrGV genome. Vertical black lines depict all sites where a nucleotide difference was observed, and red arrows show where these nucleotide changes caused a change in amino acid sequence. Predicted protein motifs are a 3C-like protease (3CLpro), nidovirus RdRp-associated nucleotidyltransferase (NiRAN), RNA-dependent RNA polymerase (RdRp), zinc-binding domain (ZBD), superfamily 1 helicase (HEL1), 3′-5′ exoribonuclease (ExoN), S-adenosylmethionine (SAM)-dependent N7- and 2′-O-methyltransferases (N-MT and O-MT, respectively) and glycoproteins (gp).
